# Is synovectomy still of benefit today in total knee arthroplasty with rheumatoid arthritis?

**DOI:** 10.1007/s00264-025-06441-3

**Published:** 2025-02-11

**Authors:** Philippe Hernigou

**Affiliations:** Paris-Est Créteil University, Créteil, France

**Keywords:** Total knee arthroplasty, Rheumatoid arthritis, Synovectomy

## Abstract

**Purpose:**

There is a lack of long-term data evaluating the impact of synovectomy versus no synovectomy during total knee arthroplasty (TKA) in patients with rheumatoid arthritis (RA). This study aimed to assess and compare bilateral TKA outcomes with and without synovectomy in the same patients over a similar follow-up period.

**Methods:**

A retrospective review was conducted on 65 bilateral staged posterior-stabilized (PS) fixed-bearing TKAs (28 men, 37 women) performed by a single surgeon on RA-affected knees, with an average follow-up of 17 years (range: 15–24 years). In the first knee, synovectomy was performed during TKA, while no synovectomy for the contralateral TKA. Outcomes assessed included Knee Society scores for knee and function, radiographic findings, complications, and patellar position using the Insall-Salvati ratio.

**Results:**

The synovectomy group had a higher rate of blood transfusion (23.3% vs. 16.6%; *P* < 0.01) and longer hospital stays (mean 9.60 days [95% CI: 6.56–13.63] vs. 6.51 days [95% CI: 5.50–9.52]; *P* < 0.001). The group without synovectomy demonstrated significantly better Knee Society Scores (89.1 vs. 80.2 points; *P* = 0.02) and greater range of motion (ROM) for flexion (130° vs. 102°; *P* = 0.01). Both groups had similar knee alignment, stability, and femoral and tibial component alignment. Patella baja was observed in six patients in the synovectomy group. Severe haematoma (*n* = 6) and deep infections (*n* = 4) were noted exclusively in the synovectomy group. Kaplan-Meier survivorship at 15 years was 81% (95% CI: 78–95) for TKA with synovectomy and 95% (95% CI: 90–100) for TKA without synovectomy.

**Conclusion:**

Knees undergoing synovectomy during primary TKA exhibited reduced knee flexion, inferior Knee Society pain scores, and higher complication rates compared to contralateral knees without synovectomy. Omitting synovectomy in RA patients did not increase the risk of implant loosening.

## Is synovectomy still benefit today in total knee arthroplasty with rheumatoid arthritis?

In rheumatoid arthritis (RA), the disease progresses from synovitis to pannus, resulting in destruction of cartilage and subchondral bone. Synovectomy was first introduced at the Society of Surgery of Paris meeting in 1900 [1]. By removing all visible synovial tissue, open synovectomy, initially proposed for the knee before the advent of arthroplasty, aimed to reduce local pain and swelling [2].

The author began performing surgeries in 1974, during a transitional period when surgical synovectomy was prominent and knee replacements in RA were just emerging. Having practiced for over half a century, the author has witnessed the evolution of synovectomy techniques, including surgical approaches using arthroscopy [3] and chemical synovectomy. Chemical synovectomy involves intra-articular injections (IAI) of glucocorticosteroids, with triamcinolone hexacetonide identified as the most effective agent, particularly in juvenile RA [4].

Radiation synovectomy, also known as radiosynoviorthesis or radiosynovectomy, uses intra-articular radionuclide injections in the form of radiolabeled colloids or particulates. First employed by Fellinger and Schmid in 1952, the most common radiopharmaceuticals have been inorganic colloids. However, leakage from the joint has sometimes led to radiation exposure in the liver, spleen, and lymph nodes. As a result, the use of radiopharmaceuticals has declined in some countries and is not recommended in others [5].

Despite its historical significance, synovectomy remains a controversial procedure. Reports have shown that many patients experienced disease progression despite undergoing synovectomy, indicating that the procedure is not universally effective. Consequently, the use of surgical and medical synovectomy for the knee has significantly diminished as a standalone treatment option.

Some surgeons in the past, before modern treatments of RA, feared that synovitis in RA may predispose to inflammatory reactions after TKA, and sometimes to loosening of arthroplasty. This has led surgeons to consider RA an indication of a synovectomy associated at the time of TKA [6–7]. Therefore, the technique involved associated full joint synovectomy to lessen the inflammatory process.

As a result, a critical aspect of the technique was performing a complete joint synovectomy to mitigate the inflammatory process, which could persist postoperatively if not thoroughly addressed as inflammatory synovitis. RA treatment strategies underwent significant evolution around the turn of this century. The utilization [8–9] of disease-modifying anti-rheumatic drugs (DMARDs) decreased surgical treatment in a substantial number of patients and improved overall health, outcome, and survival of RA patients [10–11]. Yet, little is known about how these temporal changes impact the surgical outcome in patients still in need of knee arthroplasty, particularly on the necessity of associated synovectomy, which is frequently associated with surgical complications of the TKA. There is no report with long-term FU results on the effect of synovectomy or absence of synovectomy on a TKA performed in RA. TNF-𝛼, a proinflammatory cytokine, is produced both by synovial cells and chondrocytes. Therefore, the synovectomy and chondrocyte removal performed during TKA can further enhance patient outcomes by complementing the systemic effects of DMARDs.

Therefore, the dogma of synovectomy associated with total knee replacement in rheumatoid arthritis seems never to have been questioned since. From a scientific point of view, a randomized study would undoubtedly be desirable; however, it will be challenging given the drastic decrease in indications for knee prostheses in rheumatoid arthritis [12]. For example, the author who has a large experience of TKA in RA [13] usually performed an average of eight knee TKA for polyarthritis per month in the 80–90 s decade, four per month in the 90 to 2000 period, and two per month between 2000 and 2010; and after three per year. Simultaneously, individuals using hydroxychloroquine, methotrexate, sulfasalazine or other DMARDs [11] increased twoto eightfold.

The purpose of this study was to report the experience of the author who started the absence of synovectomy for TKA in year 2000 with the agreement of the rheumatologist department and to evaluate if a synovectomy changes results of a TKA when the comparison is done with the implantation of the contralateral implant without synovectomy in the same patient and a similar followup.

## Methods

The outcomes of a consecutive series of 65 bilateral staged PS fixed-bearing total knee arthroplasties (TKAs) performed by a single surgeon on RA-affected knees over five years (2000–2004) were analyzed with a mean follow-up of 17 years (range 15–24 years). The cohort included 65 patients (28 men and 37 women), with a mean age of 44 years (range 28–53 years).

**Fig. 1:**
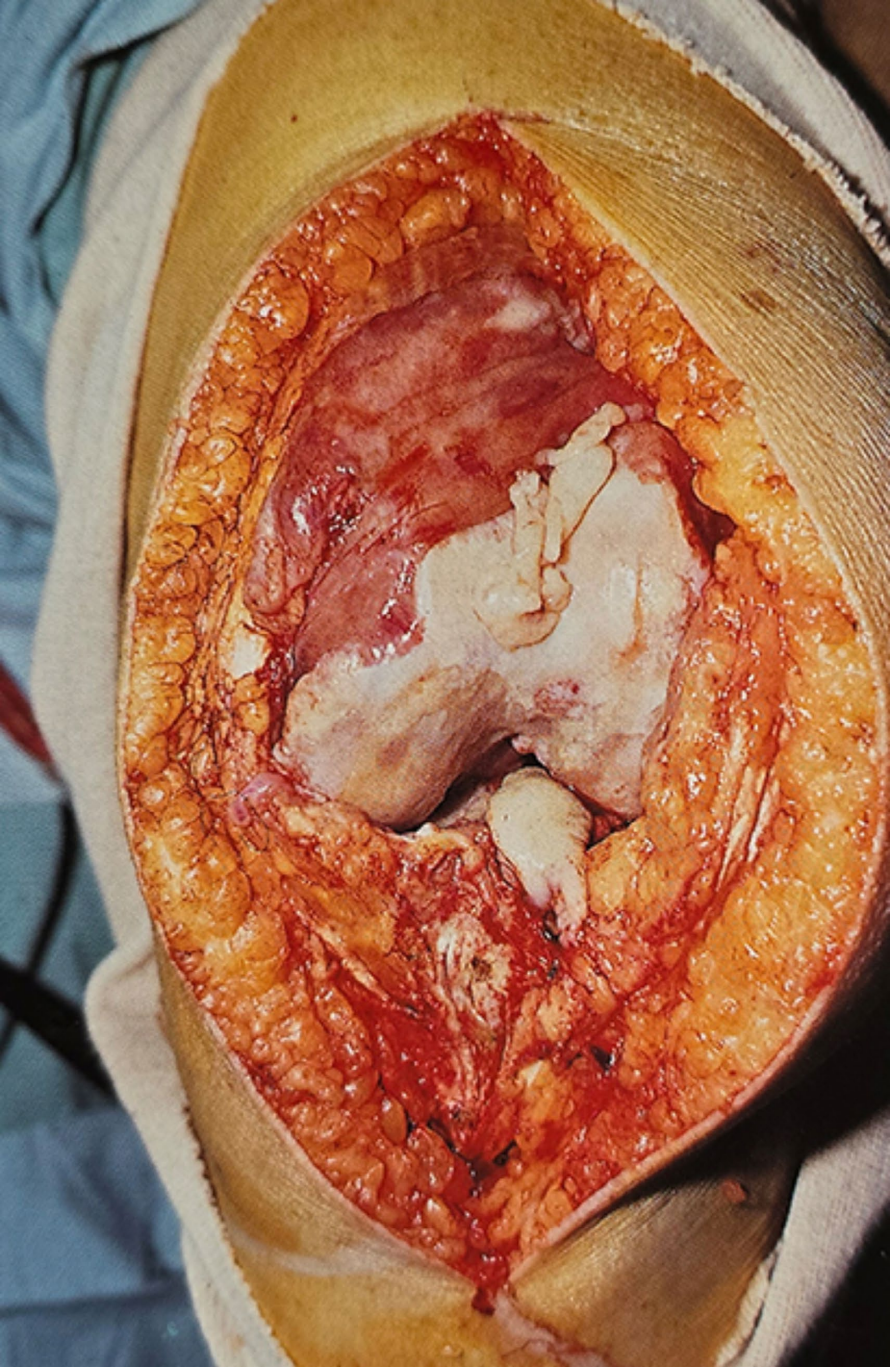
Rheumatoid arthritis knee; this view of the knee at the time of surgery shows the thickened red inflammatory pannus which extends over the surface of the femur from the suprapatellar pouch. TKA was performed without synovectomy

For the first knee, synovectomy was performed in conjunction with TKA, involving the removal of synovium from the suprapatellar and infrapatellar pouch, medial and lateral gutters, and partial excision of posterior synovium after bony resections, as complete removal was not feasible. The second knee in each patient received the same implant without synovectomy (Fig. 1), even in presence of a large pannus.

**Fig. 2:**
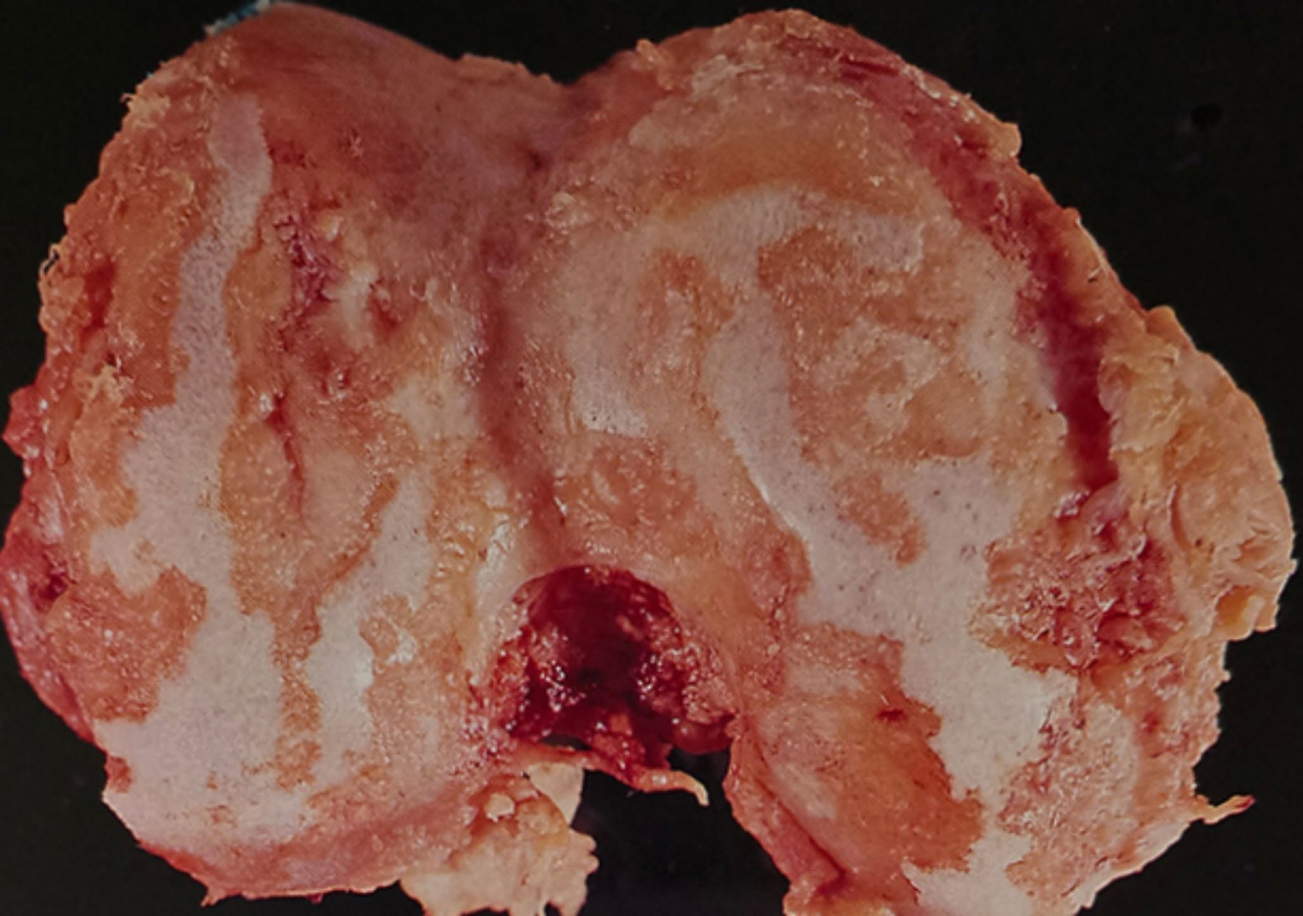
Distal femoral cut showing erosions of most of the articular cartilage of another knee where TKA was performed without synovectomy

Patients underwent clinical and radiographic evaluations, with results assessed in terms of Knee Society knee and function scores, radiographic findings, and complications. Patellar position was measured using the Insall-Salvati ratio. RA diagnoses were confirmed by the University Department of Rheumatology and corroborated during surgery through synovial fluid analysis, radiographs, and histological examination (Fig. 2). A consistent posterior-stabilized TKA implant, previously described in the literature [13], was used for all procedures.

Follow-ups were conducted at three months, one, every two years, and at the most recent followup. Standard weight-bearing anteroposterior and lateral X-rays were obtained at each visit. Pre- and postoperative evaluations, including anteroposterior and skyline radiographs, were analyzed for limb alignment, implant positioning, and the presence or location of radiolucent lines at the cement-bone interface, following Knee Society guidelines.

The aim of this article is not to describe all the DMARDs drugs associated with TKA. For each patient the treatment was not modified between the knees, and the minimum interval between two knee surgeries was six months, with a stop of 15 days before surgery and a resumption of treatment 15 days after surgery.

Pre-operation and post-operation markers (C-reactive protein [CRP], erythrocyte sedimentation rate [ESR]), pain scale (Visual Analog Scale [VAS]), infection rate, and Knee Society Score (KSS) were collected for each knee.

Statistics: for qualitative and demographic data of patients, Chi-square test was used. the nonparametric Mann-Whitney U test evaluated differences in scores between the preoperative, postoperative and latest followup. The survival rates for implants were determined using a Kaplan-Meier analysis with an endpoint of revision surgery.

## Results

The 65 Knees in the synovectomy group received blood product transfusions significantly more frequently (23.3% versus 16.6%; *P* < 0.01), had longer mean lengths of hospitalization (9.60 (95% C.I. =6.56; 13.63) days versus: 6.51 (95% C.I. =5.50; 9.52) days; *P* < 0.001). At the most recent followup differences were observed between the two groups for KSS. The Knee Score (88.1 versus 79.2 points; *p* = 0.02) and flexion (130 versus 102 degrees; *p* = 0.01) were better in the group without synovectomy.

No preoperative inflammatory marker differences could be observed since the two knees were the same patient’s. However, compared to the synovectomy group, the postoperative inflammatory markers were significantly lower in the non-synovectomy group at one week post-surgery (CRP: 35.60 ± 20.11 vs. 58.40 ± 40.13 mg/L, *p* = 0.02; ESR: 62.09 ± 13.38 vs. 80.87 ± 24.12 mm/h, *p* = 0.01) and at one month post-surgery (CRP: 12.17 ± 5.45 vs. 23.12 ± 18.12 mg/L, *p* = 0.03; ESR: 21.16 ± 18.31 vs. 31.32 ± 23.15 mm/h, *p* = 0.02), and were similar at three months after surgery. The clinical inflammatory aspect of the synovial disappeared in all the knees at six months, whether synovectomy or not.

Both groups had similar knee alignment, stability, and femoral and tibial component alignment. Patella baja was observed in six patients in the synovectomy group. Severe haematoma (*n* = 6) and deep infections (*n* = 4) were noted exclusively in the synovectomy group. Kaplan-Meier survivorship at 15 years was 81% (95% CI: 78–95) for TKA with synovectomy and 95% (95% CI: 90–100) for TKA without synovectomy.

## Discussion

Over the past three decades, significant progress has been made in understanding the pathogenesis and treatment of rheumatoid arthritis (RA). The introduction of highly effective biologic therapies has greatly reduced the prevalence of end-stage joint destruction among individuals with RA. However, despite these advancements, approximately 5–10% of patients still develop severe arthritis, with the knee being one of the most commonly affected joints, leading to pain and disability.

TKA is a proven and effective treatment for knee rheumatoid arthritis. RA is managed through a variety of medications, as nonsteroidal anti-inflammatory drugs (NSAIDs), glucocorticoids, and DMARDs. This group includes methotrexate, leflunomide, sulfasalazine, azathioprine, hydroxychloroquine, tumor necrosis factor alpha (TNF-𝛼) inhibitors, and interleukin-1 (IL-1) inhibitors.

These biologic treatments help a control of the cytokines produced that help limit proliferation of the fibroblasts, limit destruction of bone and cartilage, and the disease progression.

Inflammatory control of RA disease is critical in TKA perioperative management. When RA patients underwent elective TKA, it was recommended by the Department of Rheumatology to withhold all current biologic agents prior to surgery to prevent the risk of infection [14]. Once the wound shows evidence of healing and no non-surgical site infections occurs, the biologic therapy should be resumed. This is concordance with most of the recommendations of other series.

It is almost agreed that total synovectomy during the arthroplasty of osteoarthritis knee is not necessary. On the contrary we do not know how beneficial synovectomy in RA is in treating the pathological process in the knee compared to cartilage and bone resection alone when a TKA is performed. There was no difference in biological markers of benefit. The most important finding of the present study was that synovectomy did not improve the range of motion, pain and knee scores during the first year. On the contrary, it resulted with higher blood loss and recurrent hemarthrosis. In this study, knees with synovectomy had lower final knee flexion and lower knee scores as compared with the opposite knee of the same patient. Absence of synovectomy in rheumatoid arthritis did not increase the risk of loosening.

It was a single centre study and this is one of the limitations. The best value of this study is that surgery was performed on both knees of the same patient.

In conclusion, knees treated with complete synovectomy at the time of primary TKA had lower knee flexion and inferior KS pain scores, and more complications as compared with contralateral knees without synovectomy. Therefore, there seems no benefit for the patient in having a synovectomy performed during a knee prosthesis at this time.

## Data Availability

No datasets were generated or analysed during the current study.
